# Evaluating Collaborative Readiness for Interdisciplinary Flood Research

**DOI:** 10.1111/risa.13249

**Published:** 2018-12-11

**Authors:** Eric Tate, Valerie Decker, Craig Just

**Affiliations:** ^1^ Department of Geographical and Sustainability Sciences University of Iowa Iowa City IA USA; ^2^ Center for Evaluation and Assessment University of Iowa Iowa City IA USA; ^3^ Department of Civil and Environmental Engineering University of Iowa Iowa City IA USA

**Keywords:** Flood resilience, program evaluation, proximity

## Abstract

Increasing trends in global flood risk are driven by a complex web of interactions among natural, built environment, and social systems. As a result, flood resilience research is an ideal topic for an interdisciplinary approach. Core characteristics of interdisciplinary research are team collaboration and the systematic integration of disciplinary knowledge, in both problem formulation and analytical methods. Indicators of interdisciplinarity tend to focus on scholarly outcomes, but collaborative processes may be even more important for knowledge integration. In this Perspective piece, we outline and advocate a two‐pronged approach to enhance potential for integrating knowledge: using collaborative proximity to assess team readiness to conduct interdisciplinary research and employing program evaluation to assess change in proximity components over time. To do so, we draw on scholarship in economic geography, team science, and program evaluation. We then connect the findings to a case study of collaboration within our interdisciplinary team of flood researchers, program evaluators, and local stakeholders, as we navigate a multi‐institutional project on flood resilience.

## 1 INTRODUCTION

Global flood risk has sharply increased over recent decades, and climate change is widely expected to worsen social and economic impacts (Winsemius et al., 2016). Solutions to reduce flood risk and improve community resilience depend upon contributions from natural scientists, social scientists, and engineers, and it is thus an ideal topic to address through interdisciplinary research collaboration. Physical science contributes understanding of the role of meteorological, hydrogeological, and ecological processes. Engineering knowledge is essential to describe overland flow, the built environment, and flood extents and timing. Social science research helps explain the influence of social, economic, psychological, and political factors that may increase or reduce flood risk. The processes in each dimension operate as dynamic agents of cause, response, and moderation, to varying degrees based on place, time, and scale. Each dimension is complex independently, but even more so in the linkages among them. As a result, substantively understanding the influence that floods have on society requires an interdisciplinary approach.

As many others have observed elsewhere, interdisciplinary research can be extremely challenging, in large part because investigative teams must successfully navigate at least two major hurdles in the knowledge production process: research collaboration and doing so across academic disciplines. However, the study of scientific collaboration lags behind its practice (Bozeman & Boardman, 2014). In risk reduction research, collaboration largely remains multidisciplinary, with unrealized potential for substantive research integration (Gall, Nguyen, & Cutter, 2015; Ismail‐Zadeh, Cutter, Takeuchi, & Paton, 2017). Given the high importance of integration, it should be a core feature of the design and evaluation of interdisciplinary hazards research. Although interdisciplinary research holds great potential to advance hazards theory and reduce disaster risk, gains will be difficult to realize if collaborative processes are poorly structured to enhance knowledge integration.

In this article, we outline and advocate coupling two approaches for assessing the readiness of teams to conduct interdisciplinary hazards research: adopting collaborative proximity as an indicator of potential knowledge integration and using program evaluation to assess it. To do so, we draw on scholarship on interdisciplinary research collaboration and program evaluation and connect findings to our ongoing interdisciplinary project on flood resilience.

## 2 TEAM READINESS AND COLLABORATIVE PROXIMITY

Research collaboration is a collection of “social processes whereby human beings pool their experience, knowledge and social skills with the objective of producing new knowledge” (Bozeman & Boardman, 2014, p. 2). Studies have shown that advancing scientific understanding, addressing societally relevant problems, and personal satisfaction are primary factors that motivate researchers to participate in collaborative research (Bozeman, Fay, & Slade, 2013; Milman et al., 2015). Research into collaboration has broadly focused on characteristics of individual researchers, the composition and process of collaboration, and the organizational contexts in which it takes place (Bozeman et al., 2013). However, studies focused on the conditions leading to failed or nonproductive collaborations are scarce (Tsai, Corley, & Bozeman, 2016). The reasons underlying success and failure in interdisciplinary collaboration are even less understood.

Interdisciplinary research extends processes of research collaboration to include two additional features: collaborators from more than one academic discipline and the systematic integration of disciplinary knowledge, problem formulation, methods, and data. Conceptually, interdisciplinarity falls between multidisciplinary research with limited interaction and integration, and transdisciplinary research, which features co‐production of knowledge among academic collaborators and nonacademic stakeholders (National Research Council, 2006). Due to the combination of research collaboration and integration, interdisciplinarity is ideal for addressing complex and societally relevant issues that are too broad to be effectively investigated within a single discipline (Bark, Kragt, & Robson, 2016), such as flood risk, vulnerability, and resilience (Davidson, 2015).

Due to ease of measurement, cost, and replicability, indicators of interdisciplinarity often focus on team composition and bibliometric outcomes. For example, co‐authorship has frequently been applied as an outcome indicator of interdisciplinarity, as trends in engineering, natural sciences, and social sciences suggest movement toward more multiple authored papers (Tsai et al., 2016). However, indicators based on co‐authorship yield little understanding of the processes of knowledge generation (Bozeman et al., 2013; Wagner et al., 2011). Indeed, analysis of citation networks in resilience research suggests that substantive knowledge integration may not be occurring (Baggio, Brown, & Hellebrandt, 2015). Typical indicators of interdisciplinarity are even less applicable to *ex ante* evaluation of collaborative potential, such as in the review of interdisciplinary grant proposals (Bromham, Dinnage, & Hua, 2016).

As interdisciplinary research is fundamentally a team activity (Fiore, 2008), indicators of interdisciplinary should also measure collaborative processes. Interdisciplinarity is inherently collaborative, but places even greater importance on how knowledge exchange occurs within research teams and study designs that increase engagement (Fazey et al., 2014). Because of the higher time and resource requirements for interdisciplinary research, understanding collaborative opportunities and barriers and the readiness of teams to successfully navigate them assumes heightened importance (Armstrong & Jackson‐Smith, 2013). This in turn places greater emphasis on identifying antecedent conditions that enhance the readiness of research teams for interdisciplinarity (Hall et al., 2008).

Processes of knowledge integration and collaborative success have long been studied in the fields of economic geography and team science, using the concept of proximity. In this context, the term proximity refers to the closeness of interactions within the research collaboration environment. Proximity has been found to be a primary driver of integration capacity and innovation in collaborative settings, and it is often conceptualized in spatial, cognitive, social, and institutional dimensions (Boschma, 2005; Broekel & Boschma, 2012; Shaw & Gilly, 2000; Stokols, Harvey, Gress, Fuqua, & Phillips, 2005). We propose using these four proximity dimensions to assess the potential for knowledge integration in interdisciplinary hazards research.

*Spatial proximity* is the geographic or temporal distance among collaborators, with co‐located researchers on the near end of the proximity spectrum, and separation by large distance and/or multiple time zones on the far end. As a facilitator of interaction, spatial proximity is the principal enabler of the formation of collaboration networks and the exchange of knowledge within them (Broekel & Boschma, 2012; Shaw & Gilly, 2000). Spatial proximity can also influence the degree of continuous progress toward research goals.

*Cognitive proximity* measures the degree of overlap in knowledge bases, training, and problem conceptualization. It influences the potential for innovation, and the capacity of the team to absorb and integrate knowledge, create and adopt shared conceptualizations and analysis of the research problem, communicate effectively, and manage conflict (Gardner, 2013; Salazar, Lant, Fiore, & Salas, 2012). Conceptual modeling and visualization can be useful for identifying knowledge gaps and reaching consensus (DeLorme, Kidwell, Hagen, & Stephens, 2016; Kragt, Robson, & Macleod, 2013), particularly for navigating complexity among human and natural processes in hazards and disasters research (Reyers, Nel, O'Farrell, Sitas, & Nel, 2015).

The dimension of *social proximity* includes micro‐level social connectedness, friendship, trust, and leadership. Individually, scholars who are open‐minded, comfortable with ambiguity, and work in applied research may be best suited for participation on interdisciplinary teams (Gardner, 2013; Van Rijnsoever & Hessels, 2011). These traits can facilitate development of team‐specific norms, values, goals, communication, and trust (Kragt et al., 2013). From a project management perspective, interdisciplinary research requires greater social, organizational, and communication skills (Kragt et al., 2016), which can help bridge status hierarchies and power differentials among disciplines (MacMynowski, 2007; Salazar et al., 2012). High social proximity can help compensate for deficits in other proximity dimensions, but can also serve as a barrier to the integration of new team members. This has potentially strong implications for efforts to increase diversity and inclusion.

*Institutional proximity* is based on macro‐level rules, norms, and values that shape interdisciplinary barriers and associated individual behavior (Boschma, 2005; Thaler, Priest, & Fuchs, 2016). Here, we make a distinction between organizations (e.g., universities, departments, agencies, and nonprofits), and institutions: the formal procedures and informal customs by which organizations operate. For example, do university and departmental policies incentivize or inhibit faculty publishing in interdisciplinary journals or co‐advising graduate students across departments and colleges? When research processes and outcomes diverge from institutional norms, interdisciplinary research can be a risky endeavor, particularly for untenured faculty (Armstrong & Jackson‐Smith, 2013). It is therefore critical for research organizations to design reward structures that facilitate crossing disciplinary barriers (Gardner, 2013).

The optimal degree of proximity for knowledge integration is medium. As shown in Table I, both small and large collaborative distances in each dimension have been found to constrain research productivity (Broekel & Boschma, 2012; Cummings & Kiesler, 2008; Salazar et al., 2012; Tsai et al., 2016). Although it can be useful to conceptually separate proximity types to aid comprehension, the dimensions are interrelated (Huber, 2012). For example, spatial proximity can amplify social proximity, with close distances enhancing opportunities for interaction and building trust (Boschma, 2005). However, too much spatial proximity among collaborators may increase negative social interactions (Tsai et al., 2016). One approach to optimize proximity is to purposely build research teams with a mix of collaborative distances in each dimension (Broekel & Boschma, 2012).

**Table I:** Research Aspects Inhibited by High and Low Collaborative Proximity (Boschma, 2005)

Proximity Dimension	Inhibited by High Proximity	Inhibited by Low Proximity
Spatial	Innovation	Coordination, interaction intensity, knowledge sharing
Cognitive	Learning, novelty, creativity	Capacity to absorb, understand, and communicate concepts
Social	Entry into existing social networks, opportunism	Loyalty, level of commitment
Institutional	Awareness of opportunities, flexibility	Common habits and values

**Fig 1:**
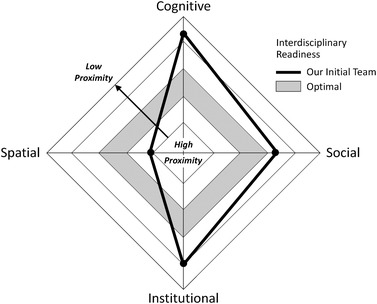
Interdisciplinary team readiness as a function of collaborative proximity.

Recent scholarship in flood risk management advocates the use of proximity to assess multiactor cooperation (Thaler et al., 2016). We propose an extension of proximity assessment into the planning, conduct, and assessment of interdisciplinary hazards research, specifically to estimate team readiness for scientific collaboration (Andrews, Newman, Meadows, Cox, & Bunting, 2012; Hays, 2008). As a tool to assess proximity, we created a radar chart to visualize the multidimensional spectrum of collaborative proximity (Fig. 1). From high values at the middle of the diamond, proximity steadily declines in each dimension to low values at the outer perimeter. The chart includes an optimal medium‐distance band shown in gray, which represents the “Goldilocks zone” of collaborative proximity between extreme values found to inhibit knowledge integration (Table I).

Fig. 1 can be used by research teams at the outset of collaboration to qualitatively assess their positioning in each proximity dimension. The results provide an estimate of team readiness to initiate and sustain interdisciplinary research. For a given dimension, a proximity value outside the optimal band highlights an area deserving deeper reflection among the team regarding potential collaborative barriers and opportunities. For spatial and institutional proximities, options for rectifying suboptimal values may be constrained due to the fixed nature of these dimensions. However, cognitive and social proximities are more malleable (Hall et al., 2008), providing a wider range of corrective actions to improve collaborative productivity.

## 3 INTERDISCIPLINARY PROGRAM EVALUATION

Fig. 1 is a simple tool for assessing collaborative readiness. A more robust approach is to embed readiness assessment into a formal program evaluation. The field of program evaluation has evolved from its roots in the 1960s to “not only to offer final judgments about the overall effectiveness of programs, but to gather process data and provide feedback to help solve programming problems along the way” (Patton, 1996, p. 12). Requests for grant proposals increasingly require a formal program evaluation to document research processes and outcomes. This pursuit takes on heightened importance for interdisciplinary research, given persistent difficulties of funding agencies in evaluating interdisciplinary projects (Bark et al., 2016). To date, program evaluation for interdisciplinarity has been most prevalent in the health sciences and for large projects (Fiore, 2008; Hall et al., 2008). We propose routine inclusion of program evaluation in proposals for interdisciplinary hazards research, to assess both research progress and collaborative processes.

Program evaluators work with the research team from the proposal stage through the final report. For interdisciplinary research, program evaluation can help clearly communicate both the work to be done and document its effectiveness. During preliminary planning, program evaluators facilitate communication and refine the research theory by applying structured frameworks such as logic models to the research goals, activities, and expected outcomes. Evaluators may also pose questions to the researchers to clarify the purpose of project components and examine the project context (Alkin, 2011). They can then design an evaluation that is feasible, useful, and responsive to stakeholder needs (Yarbrough, Shulha, Caruthers, & Hopson, 2011).

Evaluation design for internal team processes might focus on communication, the use of integrative methods, management structures, or interaction among proximity dimensions (Klein, 2008). The evaluation team could also work among the research team to identify and measure indicators of collaborative readiness, and provide ongoing monitoring of knowledge integration conditions (and suggest corrections if necessary). For example, the program evaluators might ask team members individually to describe the project goals in their own words, with the level of agreement among the team members serving as an indicator of cognitive proximity. If team members substantially disagree, this could signal an area of needed discussion.

Due to the unique role of program evaluators in research processes, the inclusion of an evaluator on the team may present additional opportunities and challenges. Program evaluation is inherently collaborative, and thus is well aligned with interdisciplinarity. However, because program evaluators do not explicitly design or implement the research itself, including a program evaluation component “can alter the programs, their contexts, and their outcomes in significant and unpredictable ways” (Yarbrough et al., 2011, p. xxvi). This is because evaluation changes research processes in aspects such as visioning, formalizing approaches, and accountability. Institutionally, program evaluators may be professional staff as opposed to faculty or graduate students. As a result, evaluator motivations and constraints for collaborative participation may differ from scientific researchers (e.g., publication expectations and freedom to contribute to grant development).

## 4 CASE STUDY: INTERDISCIPLINARY RESEARCH IN FLOOD RESILIENCE

In 2016, the U.S. Department of Housing and Urban Development awarded $97 million to the State of Iowa for its proposal, *The Iowa Watershed Approach for Urban and Rural Resilience (IWA)*. The overall project goal is to improve resilience to flood hazards at the watershed scale. Nine watersheds serve as IWA project sites, with programs to be implemented in each to increase resilience to future floods. The IWA objectives in each watershed are to reduce flood risk, improve water quality, increase flood resilience, engage stakeholders, improve quality of life (especially for socially vulnerable populations), and develop a scalable and replicable program (IWA, 2018). The IWA project provides an opportunity to illustrate proximity concepts and program evaluation in the context of interdisciplinary flood hazards research.

The IWA organizational structure includes more than a dozen funded and primarily nonacademic partners in public universities, state agencies, and city departments. The flood resilience team exists as a semiautonomous group within the larger IWA superstructure, and institutionally within a flood engineering institute at the University of Iowa. The resilience team includes two program evaluators, who focus on formative and summative evaluation for program improvement and documenting outcomes. Evaluator activities include analysis of interviews and surveys of meeting attendees, watershed planners, funded team partners, and potential users of an online geovisualization application for social resilience information (IWAIS, 2018).

The first major step for the flood resilience project was formation of the research team. How to create an interdisciplinary research team presents a bit of a chicken versus egg question: Is it best to start with a multidisciplinary group and then seek a problem to investigate, or begin with a societally relevant problem and build an interdisciplinary team to address it? For the IWA, the latter was the principal driver of team formation. The research problem involved a hazards concept (flood resilience) and scale of analysis (watershed) that were sufficiently holistic to necessitate integration across disciplines, but also sufficiently specific to allow team members to find their own problem within it.

During proposal development, proximity components heavily influenced the composition and leadership of the flood resilience team. The engineering institute selected an environmental engineer as team leader based on his experience working on interdisciplinary teams requiring community engagement, and disciplinary overlap with the engineering focus of the institute. Thus, spatial and institutional proximities between the team leader and engineering institute were high. However, the engineering institute had originally targeted a geographer as the flood resilience team leader, given his cognitive alignment with the disaster resilience focus of the proposal. But lower institutional proximity and limited schedule availability emerged as suboptimal traits for leading the team.

Fig. 1 includes estimates of the initial location of our team along each of the four proximity dimensions. In assessing the initial team readiness for interdisciplinary research, collaborative proximity was high spatially, medium‐low socially, and low cognitively and institutionally. The flood resilience team is co‐located at the University of Iowa, while external partners in city governments, state agencies, nonprofit organizations, and other universities are all within a driving distance of a few hours. Members of the flood resilience team span academic disciplines and university positions (faculty, staff, and graduate student), with the initial team including an environmental engineer, a flood resilience coordinator with a public health background, two program evaluators, two hazards geographers, and an information scientist. The university has a multidisciplinary faculty cluster initiative on water sustainability, with an institutional home in the flood engineering institute; two flood resilience team members are faculty in the cluster. Over the years, cluster activities have included colloquia, strategic planning, social gatherings, and proposal development. The cluster established a modicum of social and institutional proximity among faculty and graduate student team members at the outset of the IWA research, although connections with the evaluators, flood resilience coordinator, and information scientist were nascent.

Our main challenge early on was bridging low cognitive proximity. The team members bring knowledge of disaster resilience theory and measurement, stakeholder engagement, program evaluation, and geovisualization, but much of this expertise is mutually exclusive. We initially struggled with divergent understanding and expectations in measuring, visualizing, communicating, and improving flood resilience. For example, should disaster resilience be defined more as resistance or recovery? What degree of emphasis should be placed on constitutive physical, social, economic, and natural processes? An additional ongoing challenge has been navigating tensions between academic research and the action‐oriented and engagement goals of the IWA.

On these and other matters, the inclusion of program evaluation experts as core team members has significantly improved clarity in the research process. Selecting program evaluation methods requires robust characterization of the research framework, inputs, activities, and expected outcomes. Compared to previous interdisciplinary projects in which we have participated, the program evaluation component has compelled earlier and more explicit definition of these elements, while simultaneously building consensus and facilitating communication among the team. Overall, the evaluators have provided critical, timely, and actionable feedback about the project components and how they contribute toward identified goals and outcomes. It is unlikely that the level of cognitive alignment and progress monitoring we have achieved would have been possible without substantive investment in program evaluation as part of the original grant proposal.

However, initial misunderstanding of the evaluator role added stress to the resilience team leader. In particular, there was a misconception that program evaluation includes personnel evaluation. This highlighted that despite high spatial proximity and frequent team meetings, there was a glaring need to devote greater attention to increasing cognitive proximity. Social interaction has helped bridge this distance, for example, through discussions during meals and shared van rides during trips to and from meetings in the nine project watersheds. This development aligns with previous findings of how processes in one proximity dimension can facilitate change in another (Boschma, 2005). As voiced later by the resilience team leader:

I feel we have GOOD evaluation experts on this team. This is key. Also, I LIKE our evaluators personally. This is key as well, and a bit surprising for me. What I mean is that I assumed that evaluators would have to keep themselves at arm's length to somehow remain “independent.” If this did happen, then the van rides would be pretty stuffy and potentially awkward. I didn't know that the evaluators could/would embed themselves in the process so fully and I didn't know they would be interested in the project itself and have informed opinions about how to put it all together.

Entering year three of the five‐year flood resilience project, we have found that proximity concepts effectively explain the collaborative successes and barriers we have experienced. Spatial and institutional proximity have been essential for team formation and communication, as they bounded the potential universe of team members (Balland, 2012) and increased the ease of interaction. Cognitive and institutional proximities have been critical for the evolution of participant roles. Cognitive proximity has been the most important for internal interactions among the resilience team, while institutional proximity has been important for connections within the IWA superstructure. Social proximity has been crucial for building trust, connectedness, and mutual respect, and has functioned in synergy with other proximity dimensions. Improvements in collaborative proximity have enabled us to effectively function as a team: finding theoretical agreement, taking advantage of opportunities, navigating hurdles, and broadening our collaboration network to include additional graduate students and community stakeholders.

However, a deep understanding of collaborative processes only began to emerge after two years of frequent interaction. A core characteristic of interdisciplinary research is higher time requirements (Brown, Deletic, & Wong, 2015), time for teams to converge on a unified problem conceptualization and effectively navigate its investigation. Although we have made substantive progress in conceptualizing and assessing flood resilience and working with nonacademic stakeholders, we consider the lack of an initial readiness evaluation in our research design to be a missed opportunity. Had we been more purposeful in focusing on knowledge integration as a primary project goal, we likely would have prioritized increasing cognitive proximity at an earlier stage in the project. Ongoing readiness evaluation can also be an asset (Hall et al., 2008). Some aspects of collaboration require continued monitoring, as the team composition and internal dynamics can markedly shift during the course of a project. Failure to include readiness evaluation in the proposal budget can increase the difficulty of a mid‐course correction.

## 5 CONCLUSIONS

Flood disasters are inherently complex, societally relevant, and require solutions reflective of interactions among underlying natural, physical, social, institutional, economic, and decision‐making dimensions (Morss, Wilhelmi, Downton, & Gruntfest, 2005). As such, disaster resilience is perhaps the quintessential interdisciplinary topic. Yet, increasing calls for interdisciplinary and transdisciplinary research suggest that the demand to integrate scientific knowledge across disciplines exceeds capacity. There is a need to adjust the collaborative processes of interdisciplinary hazards research toward greater alignment with the interdisciplinary nature of hazards and disasters.

Integrating readiness assessment into interdisciplinary hazards research increases the potential for innovative and societally relevant knowledge integration. Further embedding this approach into a program evaluation may produce even more robust pathways between interdisciplinary research objectives and outcomes, while also satisfying increasing demands from funding agencies for inclusion of program evaluation. Doing so requires research teams to be purposeful about knowledge integration as a core research outcome. Fitting a research plan into a logical structure through program evaluation can facilitate intentional discussion among team members about program goals, components, and priorities. We have come to understand that interdisciplinary team functioning and knowledge integration can be improved by devoting focus throughout the research, not only to knowledge generation and outcomes, but also to the collaborative processes affecting how they will be produced.
